# Oxidative stress-dependent and -independent death of glioblastoma cells induced by non-thermal plasma-exposed solutions

**DOI:** 10.1038/s41598-019-50136-w

**Published:** 2019-09-20

**Authors:** Hiromasa Tanaka, Masaaki Mizuno, Yuko Katsumata, Kenji Ishikawa, Hiroki Kondo, Hiroshi Hashizume, Yasumasa Okazaki, Shinya Toyokuni, Kae Nakamura, Nobuhisa Yoshikawa, Hiroaki Kajiyama, Fumitaka Kikkawa, Masaru Hori

**Affiliations:** 1 Center for Low-temperature Plasma Sciences, Nagoya University, Furo-cho, Chikusa-ku, Nagoya 464-8603 Japan; 2 Center for Advanced Medicine and Clinical Research, Nagoya University Hospital, Tsurumai-cho 65, Showa-ku, Nagoya 466-8550 Japan; 3 Department of Pathology and Biological Responses, Nagoya University Graduate School of Medicine, Tsurumai-cho 65, Showa-ku, Nagoya 466-8550 Japan; 4 Department of Obstetrics and Gynecology, Nagoya University Graduate School of Medicine, Tsurumai-cho 65, Showa-ku, Nagoya 466-8550 Japan

**Keywords:** Electrical and electronic engineering, Cancer

## Abstract

Non-thermal atmospheric pressure plasma has been widely used for preclinical studies in areas such as wound healing, blood coagulation, and cancer therapy. We previously developed plasma-activated medium (PAM) and plasma-activated Ringer’s lactate solutions (PAL) for cancer treatments. Many *in vitro* and *in vivo* experiments demonstrated that both PAM and PAL exhibit anti-tumor effects in several types of cancer cells such as ovarian, gastric, and pancreatic cancer cells as well as glioblastoma cells. However, interestingly, PAM induces more intracellular reactive oxygen species in glioblastoma cells than PAL. To investigate the differences in intracellular molecular mechanisms of the effects of PAM and PAL in glioblastoma cells, we measured gene expression levels of antioxidant genes such as *CAT*, *SOD2*, and *GPX1*. Microarray and quantitative real-time PCR analyses revealed that PAM elevated stress-inducible genes that induce apoptosis such as *GADD45α* signaling molecules. PAL suppressed genes downstream of the survival and proliferation signaling network such as *YAP/TEAD* signaling molecules. These data reveal that PAM and PAL induce apoptosis in glioblastoma cells by different intracellular molecular mechanisms.

## Introduction

Non-thermal atmospheric pressure plasma is a partially ionized gas that consists of electrons, ions, radicals, and photons, and has been recently used for medical applications^1–10^. Many researchers have developed non-thermal plasma sources and found dramatic effects on sterilization^11–15^, wound healing^16–20^, blood coagulation^21–23^, and cancer treatment^24–30^. Non-thermal plasma is widely believed to induce oxidative stress in cells and tissues by producing reactive oxygen species (ROS) and reactive nitrogen species. However, interactions between plasma and biological systems are complex, and the details re dated^31–33^.

Plasma-activated solutions have been widely developed with various plasma sources and various liquids^34–36^. Thus, plasma-activated solutions have become more and more important as an option for cancer treatment. We previously developed non-thermal atmospheric pressure plasma with high electron density and applied this plasma for cancer treatments^26,37^. We showed that plasma-irradiated medium, which we called plasma-activated medium (PAM), exhibits anti-tumor effects against glioblastoma^38,39^, ovarian^40,41^, gastric^42^, pancreatic^43^, and lung cancer cells^44^. We also demonstrated that PAM induces apoptosis in glioblastoma cells by downregulating survival and proliferation signaling networks such as the Phosphoinositide 3-kinase (PI3K)/AKT signal transduction pathway^38,39^. We further developed plasma-activated Ringer’s lactate solution (PAL) for cancer treatments, and showed that PAL also induces apoptosis in glioblastoma cells^45^. However, the intracellular molecular mechanisms of cell death by each plasma-activated solution remain to be elucidated.

In this study, we compared the intracellular molecular mechanisms of cell death between PAM-treated and PAL-treated glioblastoma cells. Both PAM and PAL downregulated phospho-AKT. However, microarray analyses and quantitative real-time PCR analyses revealed differences in downstream signaling networks that are influenced by PAM and PAL. PAM upregulated gene expression of stress-inducible signaling pathways such as Growth arrest and DNA-damage-inducible protein (GADD45α) signaling to induce apoptosis. PAL downregulated gene expression of downstream signals of the survival and proliferation signaling network such as Yes-associated protein (YAP)/Transcriptional enhancer associated domain (TEAD) signaling to induce apoptosis. These results are consistent with the results that PAM induced more intracellular ROS than PAL.

## Results

### Both PAM and PAL downregulated phospho-AKT in glioblastoma cells

**Figure 1:**
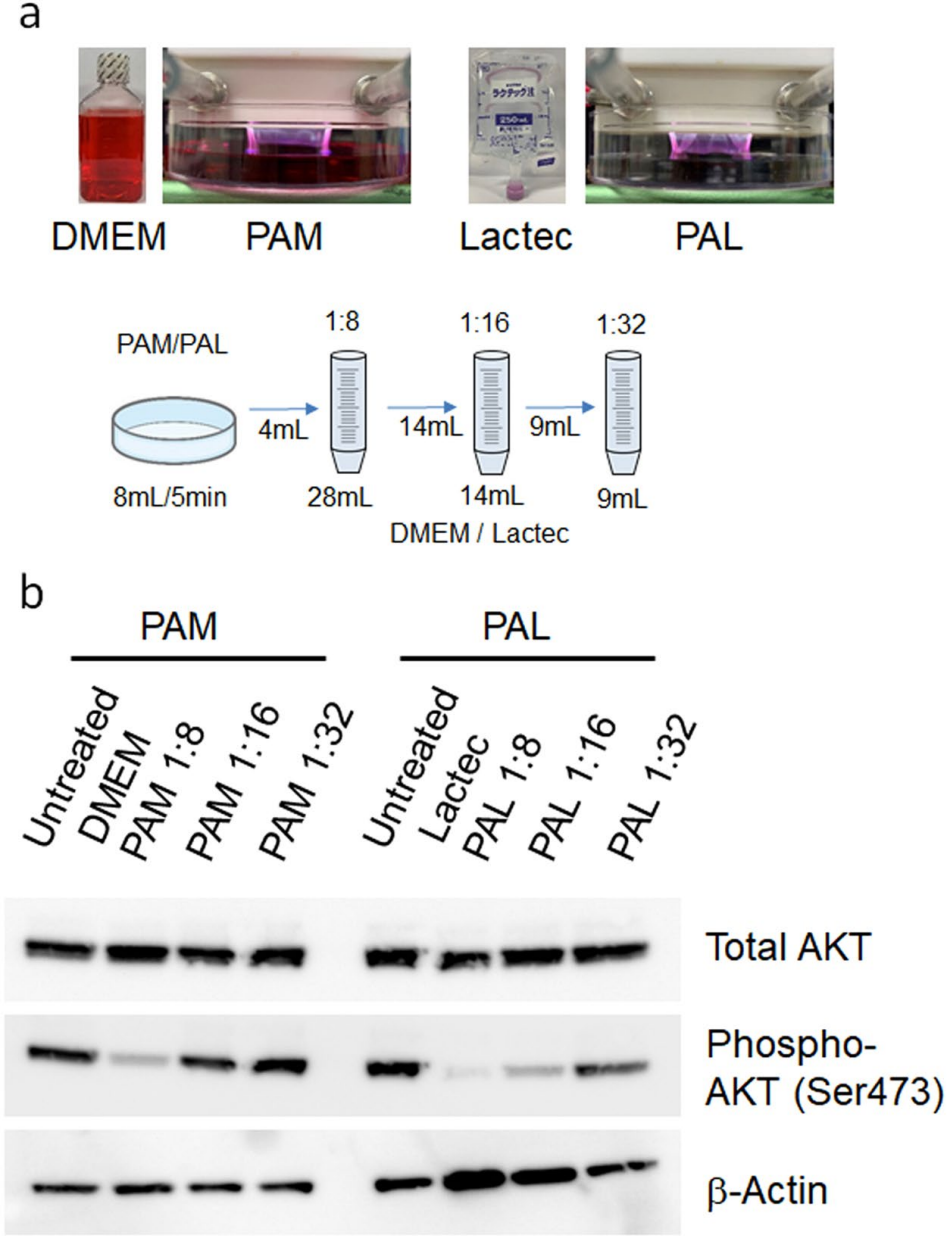
Both PAM and PAL downregulated phospho-AKT in glioblastoma cells. (**a**) Preparation of PAM and PAL and the experimental workflow. DMEM or Lactec in a 60-mm dish was treated with plasma, and PAM and PAL were diluted 8, 16, and 32 times with culture medium and Lactec, respectively. (**b**) Western blotting of total AKT and phosphorylated AKT (at Ser473) was performed on U251SP cells. β-actin was used as a loading control.

To produce PAM and PAL, 8 mL culture medium (Dulbecco’s Modified Eagle Medium; DMEM) or Ringer’s lactate solution (Lactec) was treated with plasma (the distance between the plasma source and the samples: L = 3 mm, 2.0 standard liters/min (slm)) for 5 min, as described previously^45^. PAM and PAL were diluted 8, 16, and 32 times with culture medium or Lactec, respectively, as shown in Fig. 1a. We previously reported that PAM induces apoptosis in glioblastoma cells by downregulating survival and proliferation signaling pathways including the PI3K-AKT signaling pathway^38,39^. To investigate whether PAL also affects the PI3K-AKT signaling pathway, we performed western blotting of both PAM- and PAL-treated glioblastoma cells (Fig. 1b). A range of 8-fold, 16-fold, and 32-fold dilutions of PAL downregulated phosphorylated AKT, whereas 8-fold and 16-fold dilutions of PAM downregulated phosphorylated AKT. These results suggest that PAL has a stronger effect on the PI3K-AKT signaling pathway than PAM.

### PAM induced more intracellular ROS than PAL

**Figure 2:**
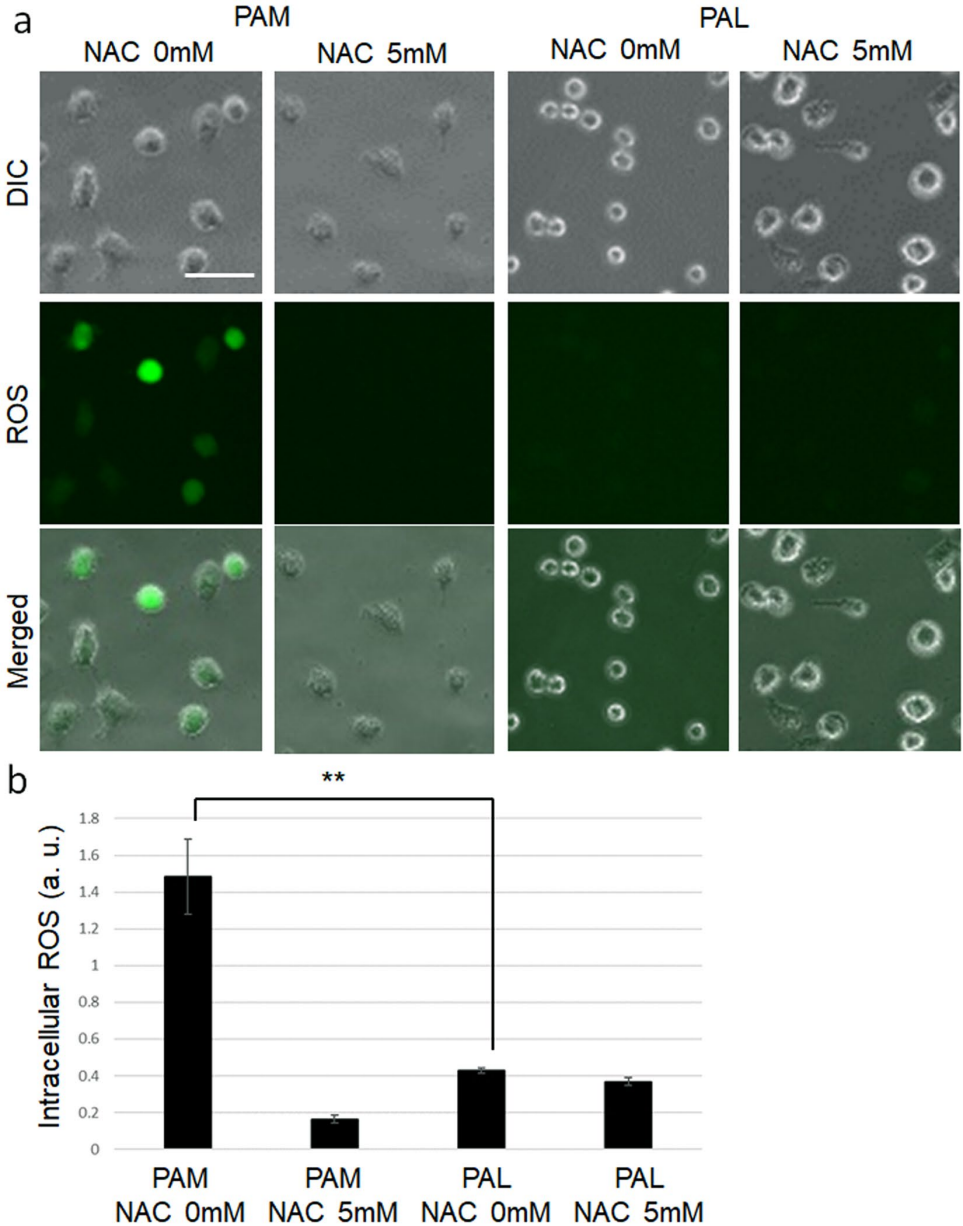
PAM- and PAL-treated glioblastoma cells with and without NAC. (**a**) Intracellular ROS generated in response to PAM and PAL. Image of U251SP cells. Scale bar represents 50 μm. DIC, differential interference contrast. (**b**) Intracellular ROS levels were evaluated by measuring fluorescent intensity of the CM-H_2_DCFDA reagent. More than 50 cells were measured. Data are the mean ± SEM. **P < 0.01 versus control.

Non-thermal plasma generally induces intracellular ROS in cells. To investigate the extent to which PAM and PAL induced intracellular ROS, we measured the fluorescent intensity of the CM-H_2_DCFDA reagent, which detects many varieties of intracellular ROS, in single cells using a fluorescence microscopy (Fig. 2a,b). To compare the intracellular ROS levels, 16-fold dilutions of PAM and PAL were used. Intracellular ROS levels in PAM-treated glioblastoma cells were significantly higher than ROS in PAL-treated glioblastoma cells. Pretreatment with 5 mM N-acetyl cysteine (NAC), a ROS scavenger, decreased intracellular ROS in PAM-treated glioblastoma cells.

**Figure 3:**
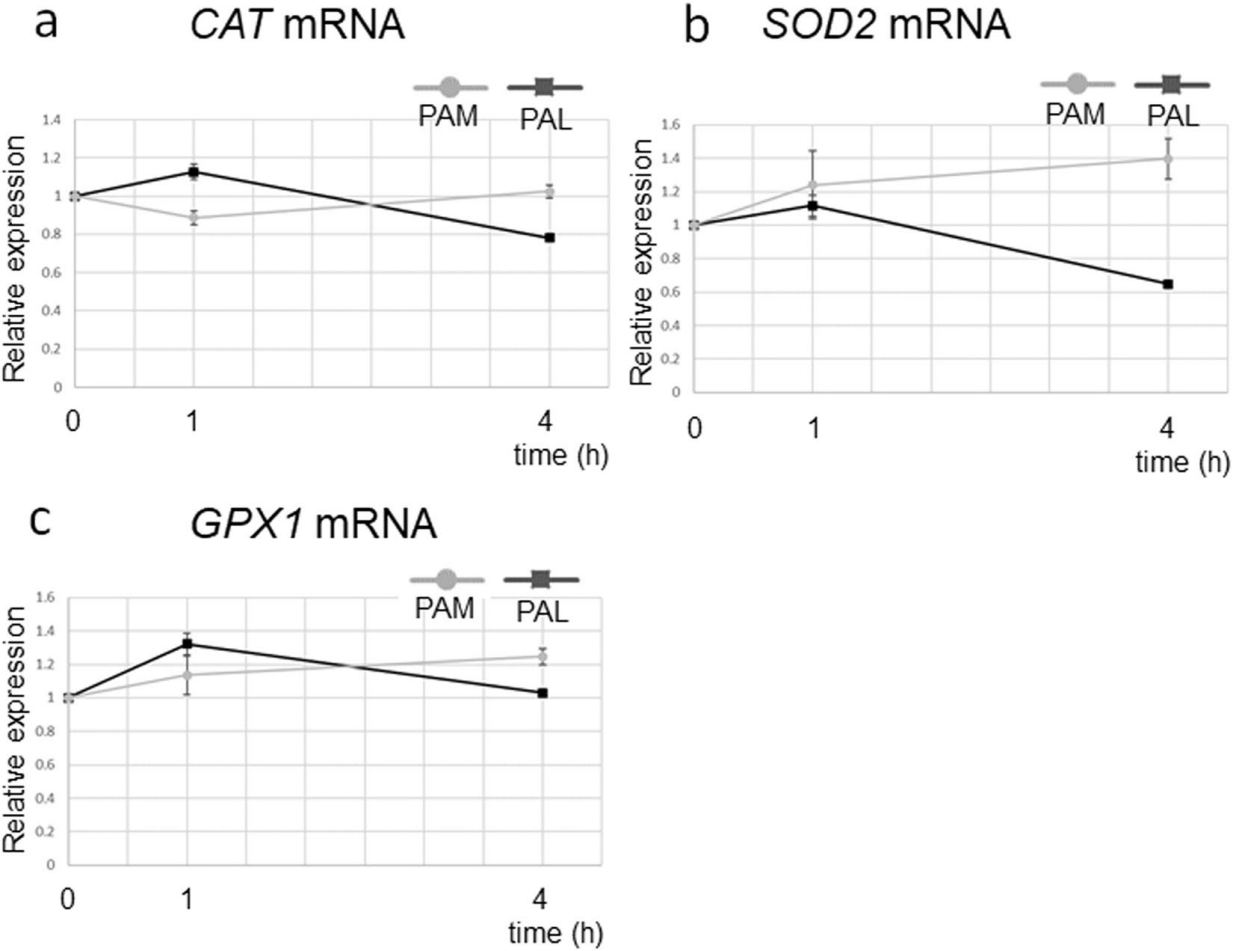
Anti-oxidant gene expression was not elevated in PAM- or PAL-treated glioblastoma cells. Relative mRNA expression of *CAT* (**a**), *SOD2* (**b**), and *GPX1* (**c**) was calculated using qRT-PCR.

To investigate gene expression of anti-oxidant genes, we performed quantitative real-time PCR (qRT-PCR) in PAM-treated and PAL-treated glioblastoma cells 1 and 4 h after PAM/PAL treatment (Fig. 3). Gene expression of the representative anti-oxidant genes, Catalase (*CAT*), Superoxide dismutase (*SOD2*), and Glutathione peroxidase (*GPX1*), was examined. Surprisingly, expression of these anti-oxidant genes was not elevated by PAM or PAL treatments. These results suggest that PAM/PAL induce other anti-oxidant genes or PAM and/or PAL induces cell death by other mechanisms.

### PAM promoted stress-related gene expression that induced apoptosis

**Figure 4:**
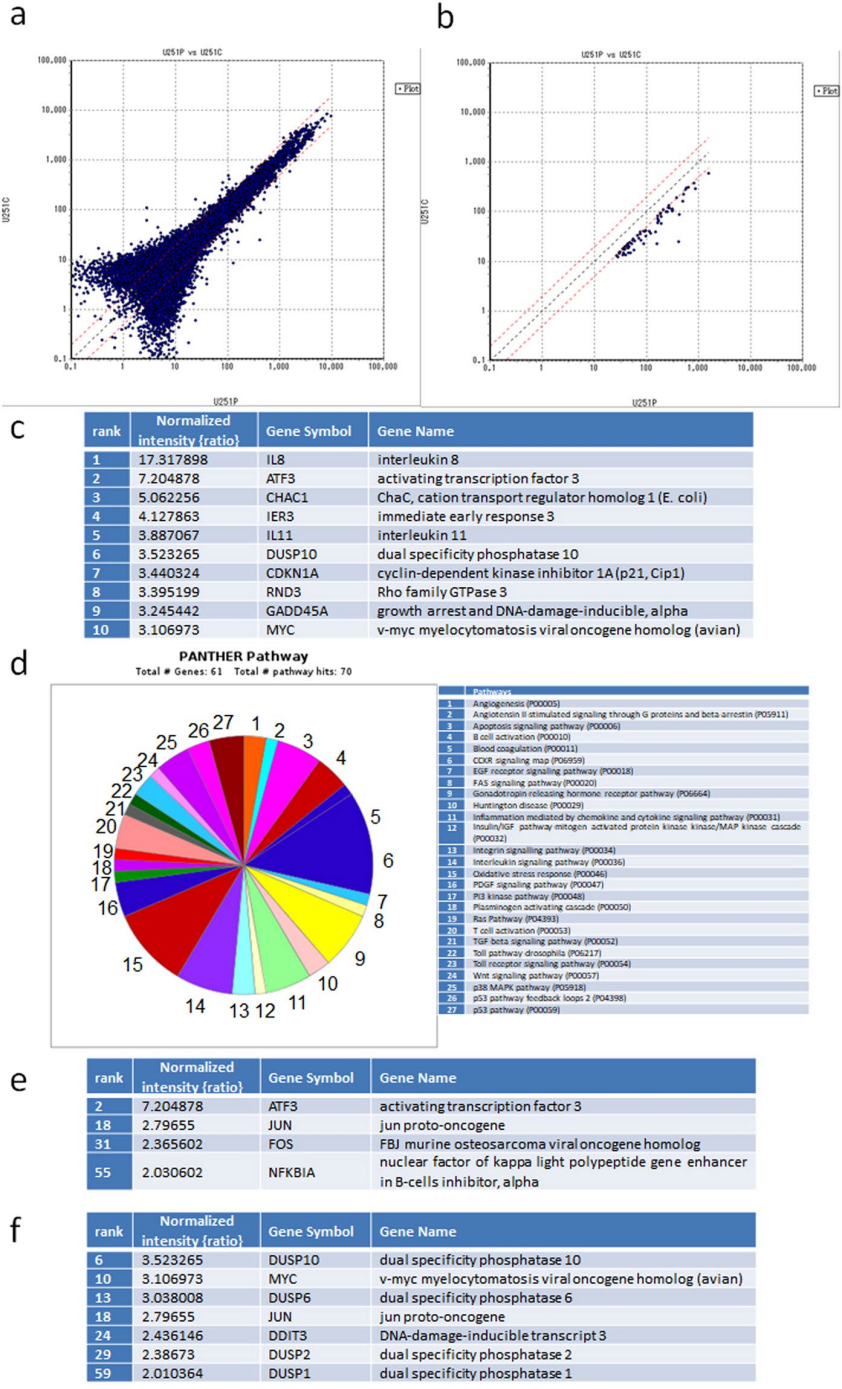
Microarray analysis revealed gene transcription networks that are activated in PAM-treated glioblastoma cells. (**a**) Gene expression profiling of PAM-treated glioblastoma cells (U251P) and untreated medium-treated glioblastoma cells (U251C) was performed using DNA microarrays. (**b**) Genes upregulated more than 2-fold in PAM-treated glioblastoma cells compared with medium-treated glioblastoma cells were selected. The cut-off value of gene expression levels of medium-treated glioblastoma cells was set at 10. (**c**) The top 10 genes upregulated in PAM-treated glioblastoma cells were ranked. (**d**) GO analyses using Panther software. We identified 61 genes that were upregulated more than 2-fold by PAM; these genes were categorized into GO terms of pathways. (**e**) Four genes that were categorized in the apoptosis signaling pathway. (**f**) Seven genes that were categorized in the oxidative stress pathway.

**Figure 5:**
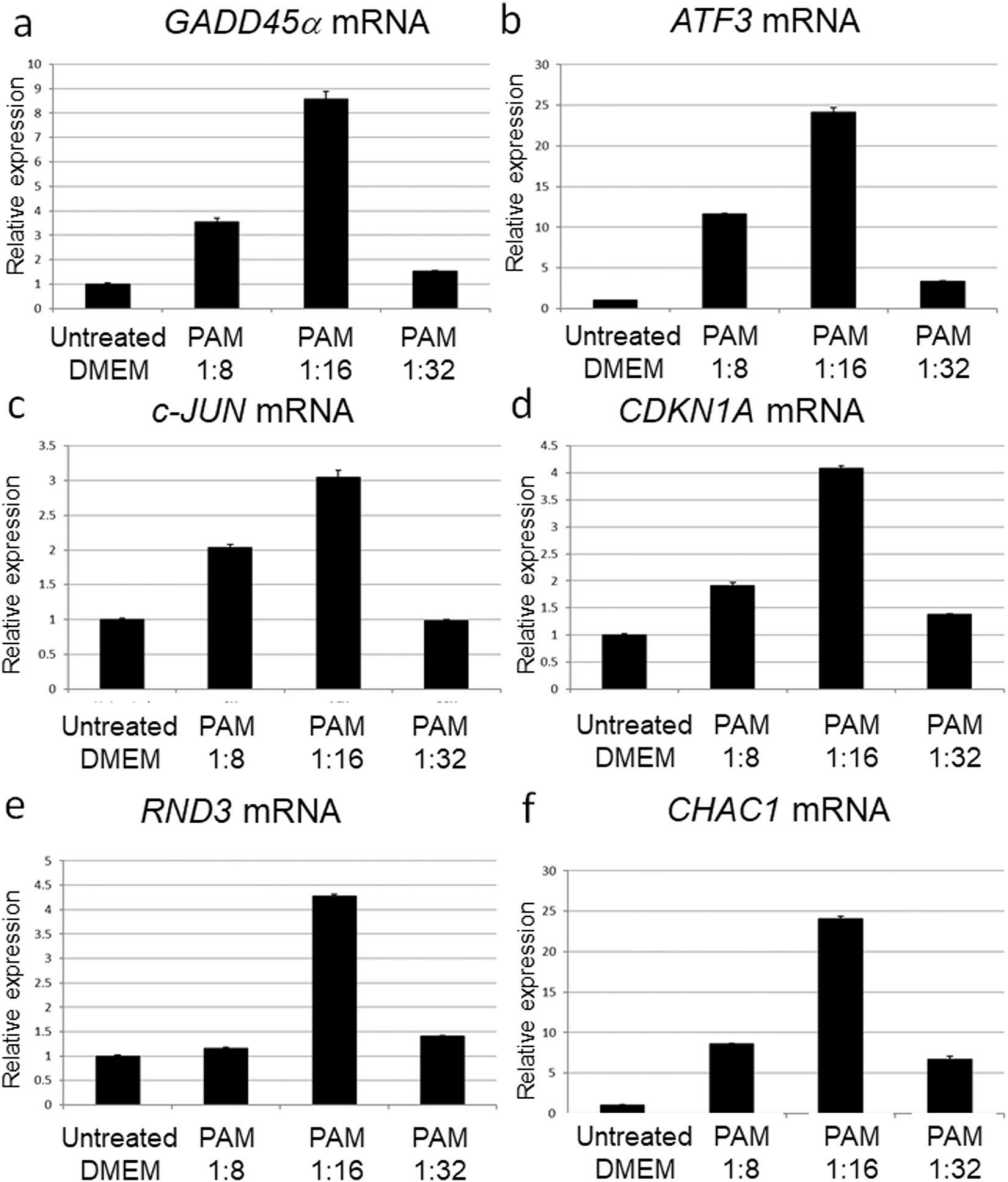
Stress-related genes that induce apoptosis were elevated in PAM-treated glioblastoma cells. Relative mRNA expression of *GADD45α* (**a**), *ATF3* (**b**), *c-JUN* (**c**), *CDKN1A* (**d**), *RND3* (**e**), and *CHAC1* (**f**) was calculated using qRT-PCR.

To investigate the gene transcription networks that are activated in PAM-treated glioblastoma cells, we performed microarray-based gene expression profiling in these cells (Fig. 4). Sixty-one genes were upregulated more than 2-fold by PAM treatment (Fig. 4a,b, Table S1). The top 10 stress-related genes that induced apoptosis included Activating transcription factor 3 (*ATF3*, rank2), Cyclin-dependent kinase inhibitor 1A (*CDKN1A*, also known as p21, rank7), and *GADD45α* (rank9) (Fig. 4c). ATF3 and c-JUN act downstream of GADD45α to mediate the stress-related pathway in glioblastoma cells^46^. Consistent with this, *c-JUN* was also upregulated by PAM (rank18, Table S1). CDKN1A also interacts with GADD45α to mediate tumor suppressor activity^47^. To validate these results, *GADD45α*, *ATF3*, *c-JUN*, and *CDKN1A* expression levels were determined with qRT-PCR (Fig. 5a–d). Glioblastoma cells were treated with 8-fold, 16-fold, and 32-fold dilutions of PAM for 2 h, and gene expression levels were measured 4 h after PAM treatment. The expression levels of these genes were correlated with each other, and the 16-fold dilution of PAM elevated these genes to the highest level. Rho family GTPase (*RND3*, also known as *RhoE*), Cation transport regulator-like protein (*CHAC1*), and Immediate early response (*IER3*, also known as *IEX1*), which also induce apoptosis in glioblastoma cells^48–50^, were ranked in the top 10 (Fig. 4c). qRT-PCR analyses showed that *RND3* and *CHAC1* were significantly upregulated by 16-fold dilution of PAM (Fig. 5e,f).

We performed gene ontology (GO) analysis of the 61 upregulated genes (Fig. 4d). Four genes (*ATF3*, *JUN*, *FOS*, and *NFKBIA*) were categorized into the term apoptosis pathway (Fig. 4e), and seven genes (*DUSP10*, *MYC*, *DUSP6*, *JUN*, *DDIT3*, *DUSP2*, and *DUSP1*) were categorized into the term oxidative stress pathway (Fig. 4f). These results suggest that PAM upregulated Dual-specificity phosphatase (DUSP) family genes to inhibit mitogen-activated protein kinases (MAPKs) through feedback regulation of MAPKs → AP-1 (c-FOS and c-JUN) → DUSP → MAPKs.

DNA damage-inducible transcript 3 (*DDIT3*), which is also known as C/EBP homologous protein (*CHOP*), is a pro-apoptotic transcription factor induced by oxidative stress, amino acid deprivation, hypoxia, and endoplasmic reticulum stress^51^. Consistent with the results in Fig. 3, anti-oxidant genes such as *CAT*, *SOD2*, and *GPX1* were not ranked among genes that were upregulated more than 2-fold in microarray analysis (Table S1).

### PAL suppressed survival- and proliferation-related gene expression

**Figure 6:**
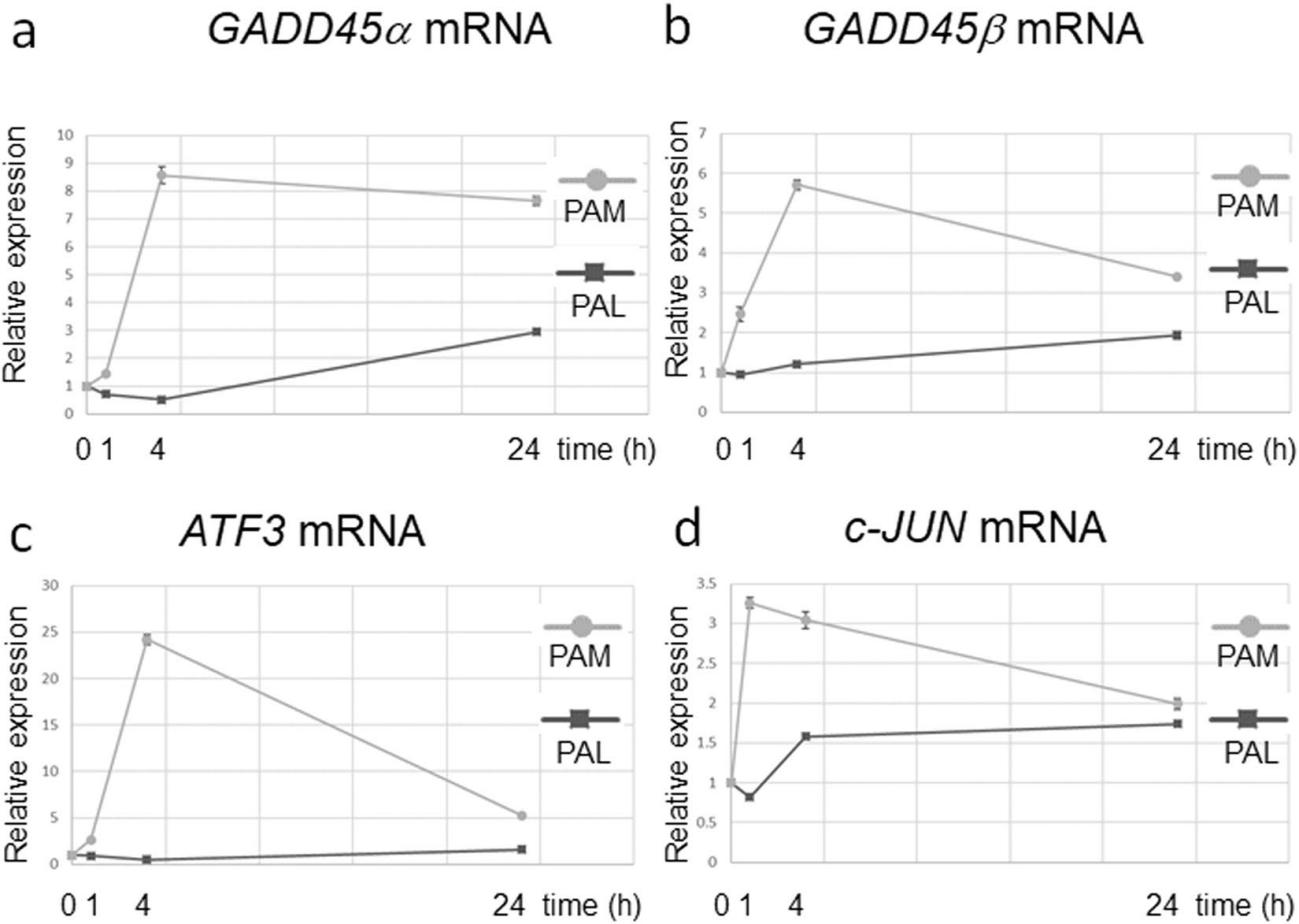
Differences in gene expression dynamics between PAM- and PAL-treated glioblastoma cells. Relative mRNA expression of *GADD45α* (**a**), *GADD45β* (**b**), *ATF3* (**c**), and *c-JUN* (**d**) was calculated using qRT-PCR.

To elucidate the different intracellular molecular mechanisms of the effects of PAM and PAL on glioblastoma cells, we investigated the dynamics of gene expression of PAM-treated and PAL-treated glioblastoma cells. We performed qRT-PCR in both PAM-treated and PAL-treated glioblastoma cells 1, 4, and 24 h after PAM/PAL treatment (Fig. 6). PAM upregulated the stress-inducible gene, *GADD45α*, and other genes related to the stress-induction pathway, whereas PAL did not greatly upregulate *GADD45α* (Fig. 6a). PAM also upregulated *GADD45β*, but PAL had a minimal effect (Fig. 6b). These results are consistent with the observation that PAM induced more oxidative stress than PAL (Fig. 2). PAL did not upregulate *ATF3* or *c-JUN*, which are downstream of *GADD45α*, (Fig. 6c,d). PAL downregulated the expression of *c-JUN* 1 h after PAL treatment.

**Figure 7:**
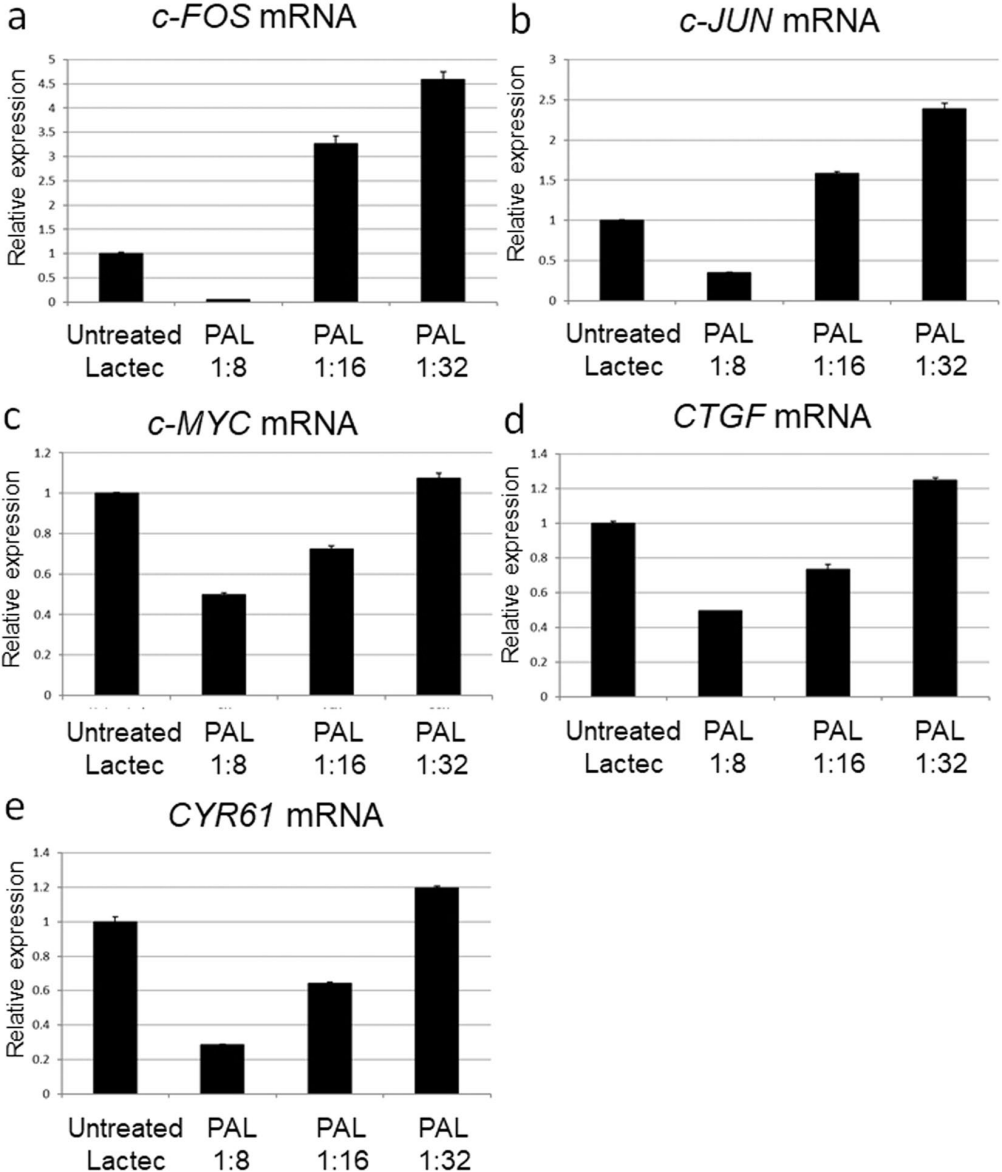
Genes downstream of the survival and proliferation signaling networks were downregulated in PAL-treated glioblastoma cells. Relative mRNA expression of *c-FOS* (**a**), *c-JUN* (**b**), *c-MYC* (**c**), *CTGF* (**d**), and *CYR61* (**e**) was calculated using qRT-PCR.

We reasoned that PAL induced apoptosis in glioblastoma cells by downregulating survival and proliferation signaling networks. Thus, we investigated the expression of genes that are downstream of the survival and proliferation signaling pathways (Fig. 7). Glioblastoma cells were treated with 8-fold, 16-fold, and 32-fold dilutions of PAL for 2 h, and gene expression levels were measured 4 h after PAL treatment. Components of the AP-1 complex, *c-FOS* and *c-JUN*, were downregulated by 8-fold dilution of PAL (Fig. 7a,b). Interestingly, genes that are downstream of YAP-TEAD signaling, including the proto-oncogene, *c-MYC*, Connective tissue growth factor (*CTGF*), and Cysteine-rich angiogenic inducer 61 (*CYR61*), were downregulated by 8-fold and 16-fold dilutions of PAL (Fig. 7c–e). These results suggest that PAL downregulated the survival and proliferation signaling networks, and are consistent with the results that PAL downregulated phospho-AKT (Fig. 1b).

## Discussion

Non-thermal plasma is believed to provide therapeutic effects by controlling the redox balance of tissues and cells. Non-thermal plasma generally produces short-lifetime and long-lifetime reactive species through interactions between plasma and air, and finally induces intracellular ROS in cells due to direct plasma treatment. PAM also induces intracellular ROS in cells through interactions among plasma, air, and liquids. Plasma interacts with components in liquids, and the physiological effects depend on the components of the plasma-activated solutions. Indeed, PAL induced less intracellular ROS than PAM (Fig. 2). These results suggest that PAL induces cell death via redox-independent mechanisms compared with PAM.

**Figure 8:**
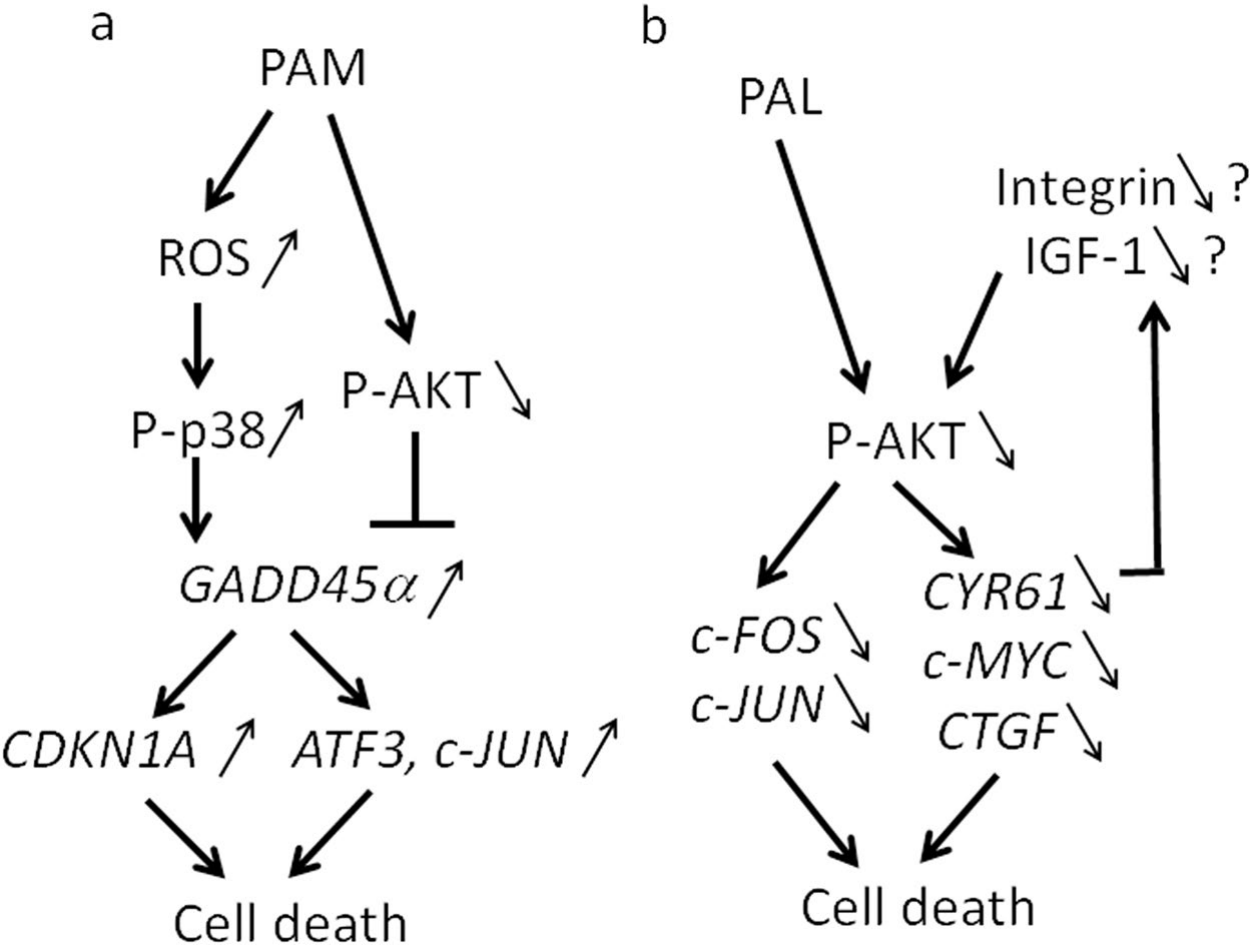
Intracellular molecular mechanisms to explain the differences between PAM- and PAL-treated glioblastoma cells. Models of intracellular molecular mechanisms of cell death in PAM-treated (**a**) and PAL-treated glioblastoma cells (**b**) based on microarray and qRT-PCR.

Our microarray and qRT-PCR analyses revealed various pathways that lead to apoptosis in PAM-treated glioblastoma cells. *GADD45α*, *ATF3*, *c-JUN*, and *CDKN1A* were consistently upregulated by PAM (Figs 4 and 5). *GADD45* family members are stress-inducible genes, and various environmental and physiological stresses such as radiation, free radicals, and pro-apoptotic cytokines upregulate *GADD45*^47,52^. Cytokine production activates a GADD45α/p38 pathway that leads to increases in ATF3 and c-JUN transcription factor levels to induce apoptosis^46^. CDKN1A is also in the GADD45/p38 signaling pathway^47^. AKT inhibition induces *GADD45α* expression in soft tissue sarcoma cells^53^. Based on these results, we elucidated the intracellular molecular mechanisms that induce apoptosis in PAM-treated glioblastoma cells (Fig. 8a). RND3, which is a Rho GTPase, inhibits cell proliferation in glioblastoma cells by interfering with Rb inactivation^48^. Temozolomide, which is a chemotherapeutic drug for treatment of glioblastoma, highly upregulates *CHAC1*, and overexpression of CHAC1 significantly influences temozolomide-mediated apoptosis in glioblastoma^49^. Overexpression of *IER3* sensitizes glioblastoma cells to γ-radiation-induced apoptosis^50^. These three genes (*RND3*, *CHAC1*, and *IER3*) were ranked in the top 10 genes that were upregulated by PAM. GO analyses revealed that PAM upregulated genes of the AP-1 complex (*FOS*, *JUN*) and DUSP genes (*DUSP1*, *DUSP2*, *DUSP6*, and *DUSP10*). DUSP family proteins are stress-induced enzymes that provide feedback inhibition of MAPKs^54^. These results suggest that PAM downregulates MAPK signaling by negative feedback through the MAPK → AP-1 → DUSP → MAPK pathway.

Gene expression analyses also revealed differences in intracellular molecular mechanisms of cell death between PAM-treated and PAL-treated glioblastoma cells. Anti-oxidant genes such as *CAT*, *SOD2*, and *GPX1* were not elevated in PAM- or PAL-treated glioblastoma cells (Fig. 3). Stress-inducible genes, such as *GADD45α/β*, *ATF3*, and *c-JUN*, which were remarkably upregulated in PAM-treated glioblastoma cells, were not upregulated by PAL (Fig. 6). On the other hand, genes downstream of the survival and proliferation signaling networks were downregulated by PAL (Fig. 7). In the U251SP glioblastoma cell line, AKT is constitutively active due to the loss of function of Phosphatase and tensin homologue deleted on chromosome ten (*PTEN*), and activated AKT protects cells from apoptosis^55^. Both PAM and PAL downregulated phospho-AKT in glioblastoma cells (Fig. 1b). The PI3K/AKT signaling pathway provides cell survival signals, in part, through activation of AP-1 transcription factors, which consist of c-FOS and c-JUN in glioblastoma cells^56^. The light-activated drug, Verteporfin, inhibits the growth of glioblastoma cells by downregulating YAP-TEAD-associated downstream signaling molecules such as c-MYC, CTGF, and CYR61^57^. CYR61 is overexpressed in glioblastoma and breast cancer cells and regulates proliferation through Integrin/Insulin-like growth factor 1 (IGF1)-AKT signaling pathways^58,59^. Based on these results, we constructed a schematic showing the putative intracellular molecular mechanisms that induce apoptosis in PAM- and PAL-treated glioblastoma cells (Fig. 8a, b respectively).

In this study, we found some differences in intracellular molecular mechanisms of cell death between PAM-treated and PAL-treated glioblastoma cells. Interestingly, PAM induced oxidative stress dependent cell death, and PAL induced oxidative stress independent cell death. These findings should be tested by *in vivo* studies. Based on our data, we can expect that we might use different plasma-activated solutions for cancers that are resistant to some plasma-activated solutions in the future.

## Methods

### Cell lines and culture

U251SP cells (human glioblastoma cell line, TP53 R273H mutation, PTEN E242fs mutation) derived at the Memorial Sloan-Kettering Cancer Institute (New York, NY)^60^ were grown in DMEM (Sigma-Aldrich, St. Louis, MO) supplemented with 10% fetal bovine serum and penicillin (100 U/mL)-streptomycin (100 μg/mL) in an atmosphere of 5% CO_2_ at 37 °C.

### Preparation of PAM and PAL

The experimental setup to prepare PAM^38^ and PAL^45^ has been previously described. While argon gas was flowing, plasma in the main discharge region was excited by applying 10 kV from a 60-Hz commercial power supply to two electrodes 20 mm apart. The flow rate of argon gas was set at 2 slm, and the distance between the plasma source and the samples was fixed at L = 3 mm. Eight milliliters DMEM or Lactec in a 60-mm dish was treated with plasma (L = 3 mm, 2.0 slm), and then PAM and PAL were diluted 8, 16, and 32 times with culture medium and Lactec, respectively (Fig. 1). These PAM and PAL were immediately used for experiments after preparation. The cell viability of U251SP cell lines was measured to test the reproducibility of PAM and PAL as previously described^38,45^.

### Western blot

Glioblastoma cells (approx. 300,000) were seeded in 3 mL medium in a six-well plate. On the following day, the medium of the cells in the six-well plate was replaced with 3 mL freshly prepared PAM or PAL. After 2 h, PAM and PAL were replaced with 3 mL culture medium. Two hours later, cells were collected, cell lysates were prepared, and western blotting was performed as previously described^38^. Western blotting for total AKT and phosphorylated AKT (at Ser473) was performed on U251SP cells. β-actin was used as a loading control.

### Detection of intracellular ROS

U251SP cells (10,000) were seeded in an eight-well chamber slide in 200 μL culture medium. On the following day, the medium of the cells in the eight-well chamber slide was replaced with 200 μL CM-H_2_DCFDA (Life Technologies, Carlsbad, CA) (10 μM) in PBS with and without 5 mM NAC (Sigma-Aldrich). After 1 h, 200 μL CM-H_2_DCFDA with and without NAC in the cell culture chambers was replaced with freshly prepared 16 times diluted PAM or PAL. After 2 h, PAM and PAL were replaced with 200 μL culture medium. After 2 h, the cells were observed using a BZ9000 microscope (Keyence, Osaka, Japan).

### Microarray

Glioblastoma cells (300,000) were seeded in 3 mL medium in a six-well plate. On the following day, 4 mL culture medium in a 60 mm-dish was treated with plasma (L = 5 mm, 2.0 slm) and the medium of the cells in the six-well plate was replaced with 3 mL PAM. After 2 h, PAM was replaced with 3 mL culture medium. After 2 h, total RNA from PAM-treated cells was isolated using an RNeasy Mini Kit (QIAGEN, Hilden, Germany) according to the manufacturer’s protocol. RNA (1 μg) was labeled with Cy3 and then hybridized with CodeLink Human Whole Genome Bioarray (Applied Microarrays, Tempe, AZ) and scanned with a microarray scanner GenePix4000B (Olympus, Kyoto, Japan). Raw intensity measurements of all probe sets were background-corrected, normalized, and converted into expression measurements using the MicroArray Data Analysis Tool Version 3.2 (Filgen, Nagoya, Japan). GO analysis was performed using the PANTHER Classification System Resource 14.0 online software (http://pantherdb.org/).

### qRT-PCR

Glioblastoma cells (300,000) were seeded in 3 mL medium in a six-well plate. On the following day, 8 mL culture medium or Lactec in a 60-mm dish was treated with plasma (L = 3 mm, 2.0 slm), and PAM and PAL were diluted 8, 16, and 32 times with culture medium and Lactec, respectively. The medium of the cells in the six-well plate was replaced with 3 mL PAM or PAL. After 2 h, PAM and PAL were replaced with 3 mL culture medium. One, four, and twenty-four hours after PAM or PAL treatment, RNA from PAM- and PAL-treated cells was extracted using the RNeasy Mini Kit (QIAGEN) according to the manufacturer’s protocol. Reverse transcription was performed using the Omniscript RT Kit (QIAGEN) to synthesize cDNA. qRT-PCR was conducted using KOD SYBR qPCR Mix (TOYOBO, Osaka, Japan) and monitored in real-time using the LightCycler®480 PCR system (Roche Diagnostics, Rotkreuz, Switzerland). Relative mRNA expression was calculated using the 2^−ΔΔCT^ method. Expression of all target genes was normalized to GAPDH as a reference. Primers used in this study are described in Table 1. All PCR analyses were performed in triplicate.

**Table 1:** The sequences of primers used for qRT-PCR.

Target gene	Sequence
CAT	F′: 5′- GGTCATGCATTTAATCAGGCAGAA -3′
	R′: 5′- TTGCTTGGGTCGAAGGCTATC -3′
SOD2	F′: 5′- CCAAATCAGGATCCACTGCAA -3′
	R′: 5′- CAGCATAACGATCGTGGTTTACTT -3′
GPX1	F′: 5′- CAGTTGCAGTGCTGCTGTCTC -3′
	R′: 5′- GCTGACACCCGGCACTTTATTAG -3′
GADD45α	F′: 5′- CTGCAGTTTGCAATATGACTTTGG -3′
	R′: 5′- GGGCTTTGCTGAGCACTTC -3′
GADD45β	F′: 5′- CGAGTCGGCCAAGTTGATGA -3′
	R′: 5′- ACCCGCACGATGTTGATGTC -3′
ATF3	F′: 5′- ACCAGGATGCCCACCGTTAG -3′
	R′: 5′- GACAATGGTAGCCACGGTGAAG -3′
c-JUN	F′: 5′- ACCAAGAACTGCATGGACCTAACA -3′
	R′: 5′- GCTCAGCCTCGCTCTCACAA -3′
CDKN1A	F′: 5′- CATGTGGACCTGTCACTGTCTTGTA -3′
	R′: 5′- ATCTTCAAGGAGCGTCACCACAC -3′
RND3	F′: 5′- TCATGGATCCTAATCAGAACGTGAA -3′
	R′: 5′- GAAGTGTCCCACAGGCTCAACTC -3′
CHAC1	F′: 5′- GTTTCTGGCAGGGAGACACCTT -3′
	R′: 5′- ATCTTCAAGGAGCGTCACCACAC -3′
c-FOS	F′: 5′- TCTTACTACCACTCACCCGCAGAC -3′
	R′: 5′- GGAATGAAGTTGGCACTGGAGAC -3′
c-MYC	F′: 5′- CCTGGTGCTCCATGAGGAGA -3′
	R′: 5′- CAGTGGGCTGTGAGGAGGTTT -3′
CTGF	F′: 5′- CTTGCGAAGCTGACCTGGAA -3′
	R′: 5′- AAAGCTCAAACTTGATAGGCTTGGA -3′
CYR61	F′: 5′- CCAAGCAGCTCAACGAGGA -3′
	R′: 5′- TGATGTTTACAGTTGGGCTGGAA -3′
GAPDH	F′: 5′- CGCTCTCTGCTCCTCCTGTTC -3′
	R′: 5′- ATCCGTTGACTCCGACCTTCAC -3′

### Statistical analysis

All data are presented as the mean ± the standard error of the mean (SEM). The unpaired Student’s t-test (two-tailed) was used.

### Comments

By submitting a comment you agree to abide by our Terms and Community Guidelines. If you find something abusive or that does not comply with our terms or guidelines please flag it as inappropriate.

## Supplementary information

Supplemental Information

## Data Availability

The data that support the findings of this study are available from the corresponding author upon reasonable request.
