# Supplementary material for: Oxidative stress-dependent and -independent death of glioblastoma cells induced by non-thermal plasma-exposed solutions
Source: Sci Rep. 2019 Sep 20;9:13657. doi: 10.1038/s41598-019-50136-w (PMC6754505; doi:10.1038/s41598-019-50136-w)
Supplement: Supplementary file 1 — Supplemental Information [file 41598_2019_50136_MOESM1_ESM.pdf]

Oxidative stress-dependent and -independent death of glioblastoma cells induced by non-thermal plasma-exposed solutions

Hiromasa Tanaka[1, 2 *], Masaaki Mizuno[2], Yuko Katsumata[1], Kenji Ishikawa[1], Hiroki Kondo[1], Hiroshi Hashizume[1], Yasumasa Okazaki[3], Shinya Toyokuni[3], Kae Nakamura[4], Nobuhisa Yoshikawa[4], Hiroaki Kajiyama[4], Fumitaka Kikkawa[4], and Masaru Hori[1]

[1] Center for Low-temperature Plasma Sciences, Nagoya University, Furo-cho, Chikusa-ku, Nagoya 464-8603, Japan

[2] Center for Advanced Medicine and Clinical Research, Nagoya University Hospital, Tsurumai-cho 65, Showa-ku, Nagoya 466-8550, Japan

[3] Department of Pathology and Biological Responses, Nagoya University Graduate School of Medicine, Tsurumai-cho 65, Showa-ku, Nagoya 466-8550, Japan

[4] Department of Obstetrics and Gynecology, Nagoya University Graduate School of Medicine, Tsurumai-cho 65, Showa-ku, Nagoya 466-8550, Japan

*To whom correspondence should be addressed.

E-mail: htanaka@plasma.engg.nagoya-u.ac.jp

Table S1: List of the 61 genes that were upregulated more than 2-fold by PAM.

rank	Normalized intensity {ratio}	Gene Symbol	Gene Name
1	17.317898	IL8	interleukin 8
2	7.204878	ATF3	activating transcription factor 3
3	5.062256	CHAC1	ChaC, cation transport regulator homolog 1 (E. coli)
4	4.127863	IER3	immediate early response 3
5	3.887067	IL11	interleukin 11
6	3.523265	DUSP10	dual specificity phosphatase 10
7	3.440324	CDKN1A	cyclin-dependent kinase inhibitor 1A (p21, Cip1)
8	3.395199	RND3	Rho family GTPase 3
9	3.245442	GADD45A	growth arrest and DNA-damage-inducible, alpha
10	3.106973	MYC	v-myc myelocytomatosis viral oncogene homolog (avian)
11	3.087178	CTGF	connective tissue growth factor
12	3.080271	SNAI2	snail homolog 2 (Drosophila)
13	3.038008	DUSP6	dual specificity phosphatase 6
14	3.018653	GADD45B	growth arrest and DNA-damage-inducible, beta
15	3.010193	MEFV	Mediterranean fever
16	2.851969	LITAF	lipopolysaccharide-induced TNF factor
17	2.806354	CYR61	cysteine-rich, angiogenic inducer, 61
18	2.79655	JUN	jun proto-oncogene
19	2.584015	COL11A1	collagen, type XI, alpha 1
20	2.498025	PTAFR	platelet-activating factor receptor
21	2.481692	ZFP36	zinc finger protein 36, C3H type, homolog (mouse)
22	2.457079	RASSF4	Ras association (RalGDS/AF-6) domain family member 4
23	2.452987	HMGA2	high mobility group AT-hook 2
24	2.436146	DDIT3	DNA-damage-inducible transcript 3
25	2.408367	ERRFI1	ERBB receptor feedback inhibitor 1
26	2.405796	SEMA7A	semaphorin 7A, GPI membrane anchor (John Milton Hagen blood group)
27	2.39901	PLK3	polo-like kinase 3
28	2.388136	GYPA	glycophorin A (MNS blood group)
29	2.38673	DUSP2	dual specificity phosphatase 2
30	2.372008	HBEGF	heparin-binding EGF-like growth factor

31	2.365602	FOS	FBJ murine osteosarcoma viral oncogene homolog
32	2.3536	IFITM5	interferon induced transmembrane protein 5
33	2.33729	KLF6	Kruppel-like factor 6
34	2.281947	THOC2	THO complex 2
35	2.233788	GCM1	glial cells missing homolog 1 (Drosophila)
36	2.233557	EGR1	early growth response 1
37	2.210247	A1BG	alpha-1-B glycoprotein
38	2.183498	TRIB1	tribbles homolog 1 (Drosophila)
39	2.179448	PLAU	plasminogen activator, urokinase
40	2.140145	ITFG1	integrin alpha FG-GAP repeat containing 1
41	2.128974	ABL2	v-abl Abelson murine leukemia viral oncogene homolog 2
42	2.124016	SLC30A1	solute carrier family 30 (zinc transporter), member 1
43	2.102962	LBX1	ladybird homeobox 1
44	2.093884	VAMP5	vesicle-associated membrane protein 5 (myobrevin)
45	2.0832	SCLY	selenocysteine lyase
46	2.080763	RUNX1-IT1	RUNX1 intronic transcript 1 (non-protein coding)
47	2.073478	LTBP2	latent transforming growth factor beta binding protein 2
48	2.067477	HMOX1	heme oxygenase (decycling) 1
49	2.056835	IER2	immediate early response 2
50	2.056613	ABL2	v-abl Abelson murine leukemia viral oncogene homolog 2
51	2.045163	UTS2R	urotensin 2 receptor
52	2.037542	DIDO1	death inducer-obliterator 1
53	2.032995	ZFP36L1	zinc finger protein 36, C3H type-like 1
54	2.031219	SGK1	serum/glucocorticoid regulated kinase 1
55	2.030602	NFKBIA	nuclear factor of kappa light polypeptide gene enhancer in B-cells inhibitor, alpha
56	2.028303	SERTAD1	SERTA domain containing 1
57	2.0219	SORBS2	sorbin and SH3 domain containing 2
58	2.016448	CLDN10	claudin 10
59	2.010364	DUSP1	dual specificity phosphatase 1
60	2.006636	STC2	stanniocalcin 2
61	2.000084	EAF1	ELL associated factor 1

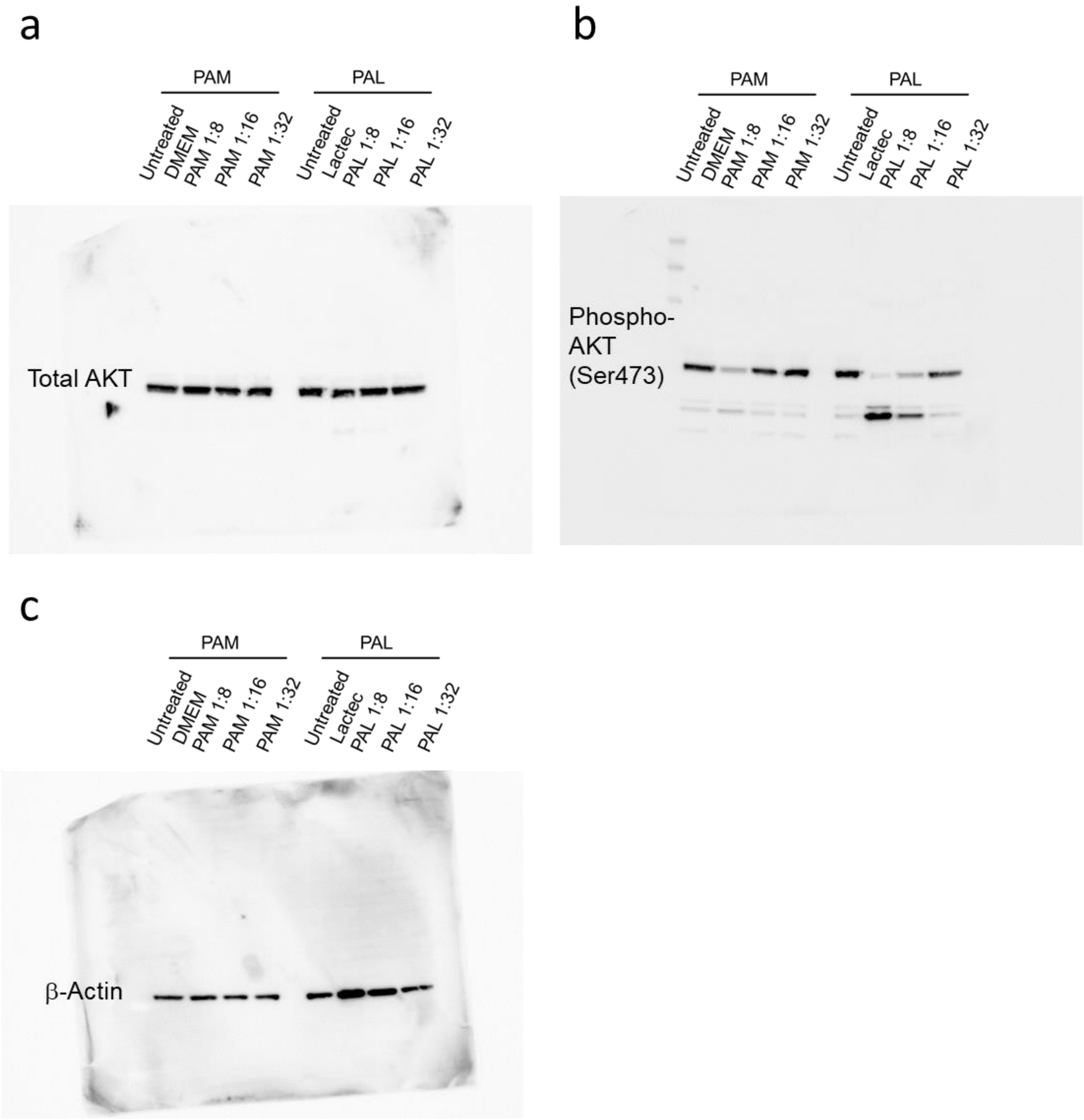

Figure S1: Full length blots of Figure 1
